# Analogue Play in the Age of AI: A Scoping Review of Non-Digital Games as Active Learning Strategies in Higher Education

**DOI:** 10.3390/bs16010133

**Published:** 2026-01-16

**Authors:** Elaine Conway, Ruth Smith

**Affiliations:** 1 Loughborough Business School, Loughborough University, Loughborough LE11 3TU, UK; 2 School of Business, University of Leicester, Leicester LE2 1RQ, UK

**Keywords:** active learning, analogue games, board games, card games, higher education, game-based learning, generative artificial intelligence, scoping review

## Abstract

Non-digital traditional games such as board and card formats are increasingly recognised as valuable tools for active learning in higher education. These analogue approaches promote engagement, collaboration, and conceptual understanding through embodied and social interaction. This scoping review mapped research on the use of traditional, non-digital games as active learning strategies in tertiary education and examined whether the rise in generative artificial intelligence (GenAI) since 2022 has influenced their pedagogical role. Following the PRISMA-ScR framework, a systematic search of Scopus (October 2025) identified 2480 records; after screening, 26 studies met all inclusion criteria (explicitly using card and/or board games). Whilst this was a scoping, not a systematic review, some bias due to using only one database and evidence could have missed some studies. Results analysed the use and impacts of the games and whether AI was a specific driver in its use. Studies spanned STEM, business, health, and social sciences, with board and card games most frequently employed to support engagement, understanding, and collaboration. Most reported positive learning outcomes. Post-2023 publications suggest renewed interest in analogue pedagogies as authentic, human-centred responses to AI-mediated education. While none directly investigated GenAI, its emergence appears to have acted as an indirect catalyst, highlighting the continuing importance of tactile, cooperative learning experiences. Analogue games therefore remain a resilient, adaptable form of active learning that complements technological innovation and sustains the human dimensions of higher-education practice.

## 1. Introduction

### 1.1. Active Learning and the Pedagogical Value of Play

Active learning has become a defining principle of contemporary higher education, emphasising student participation, collaboration, and critical engagement rather than passive knowledge transmission (Prince, 2004; Freeman et al., 2014). Within this paradigm, educators have increasingly adopted playful and game-based approaches to promote interaction, motivation, and problem-solving (Deterding et al., 2011; Hamari et al., 2016). Game-based learning (GBL) strategies, ranging from simulations to role-play and structured competitions, are valued for their ability to cultivate deep learning through authentic, experiential contexts (Gee, 2008). They have been widely adopted in higher education to support student motivation, engagement, and active participation, with systematic reviews demonstrating their pedagogical potential across diverse disciplines (Dicheva et al., 2015; Subhash & Cudney, 2018).

However, most literature on GBL has focused on digital environments, reflecting the rapid expansion of educational technology over the past two decades. Digital games and virtual platforms have dominated discussions of engagement and interactivity in higher education, often eclipsing the continued relevance of non-digital, or analogue, approaches. However, long before digital gamification became ubiquitous, educators used board and card games to simulate complex systems, teach procedural knowledge, and encourage reflective learning through embodied, social experience (Lean et al., 2006; Gentry et al., 2019). Recent studies indicate a resurgence of such analogue tools, particularly in disciplines seeking to balance cognitive challenge with creativity and interpersonal learning (Rafiq et al., 2025; Conway & Smith, 2026).

### 1.2. The Case for Analogue Game-Based Learning and Active Learning

Analogue games, such as board, card, or similar tabletop formats, offer distinctive pedagogical affordances that differ from their digital counterparts. They are inherently embodied, requiring learners to manipulate materials, read social cues, and negotiate meaning collaboratively. The physical presence of peers and artefacts promotes social learning (Vygotsky, 1978) and distributed cognition (Hutchins, 1995), making them powerful tools for developing interpersonal and metacognitive skills.

Research has shown that non-digital GBL supports a wide range of outcomes in higher education, including improved conceptual understanding (Martindale & Weiss, 2020), enhanced teamwork and communication (Ferreira Dias et al., 2024), and increased learner motivation (Sotoca-Orgaz et al., 2025). These games also provide low-technology, inclusive options for institutions or disciplines with limited access to advanced digital infrastructure. Their design simplicity encourages adaptation across subjects, from chemistry and engineering to business, health, and the social sciences.

Despite these advantages, analogue games remain under-represented in the educational research literature. Most existing reviews either conflate digital and non-digital games or prioritise technology-enhanced learning (Subhash & Cudney, 2018). Consequently, there is limited synthesis of how traditional, physical games are currently employed in higher education, what learning benefits they generate, and how their role may be shifting within a rapidly digitising academic environment. However, these analogue activities provide tactile, social, and collaborative experiences that align with principles of active learning and experiential pedagogy. They encourage learners to construct understanding through participation and reflection rather than passive reception, supporting critical thinking, teamwork, and communication.

The rise in GenAI has intensified debates around what constitutes meaningful learning in higher education (Kasneci et al., 2023; Knox, 2023; Selwyn et al., 2023). With AI systems increasingly capable of reproducing factual knowledge and even simulating reasoning (Zawacki-Richter et al., 2019; Holmes et al., 2019), educators are re-evaluating the value of embodied, social, and creative learning (Luckin et al., 2016). Active learning, where students engage collaboratively in constructing understanding, has therefore become both a pedagogical necessity and a response to automation (Topali et al., 2025). Within this context, non-digital games offer tangible, interactive experiences that foreground the human dimensions of cognition and collaboration (Jandrić et al., 2018). They provide opportunities for authentic engagement, ethical reasoning, and collective problem-solving that cannot easily be replicated through AI-mediated environments.

Consequently, active learning was adopted as the central search term in this review to identify pedagogical practices explicitly designed to promote learner participation and knowledge co-construction within higher education settings.

### 1.3. GenAI and the Changing Context of Learning

The educational landscape has changed profoundly since the public release of ChatGPT in November 2022, which marked the mainstream introduction of GenAI to higher education. Within months, institutions worldwide confronted new questions about academic integrity, authorship, and the nature of learning itself (Zawacki-Richter et al., 2019). Whereas earlier forms of educational AI were primarily analytical, focused on adaptive assessment or learning analytics, GenAI tools generate text, images, and code that can replicate or replace cognitive work traditionally performed by students (Kasneci et al., 2023). Hence, whilst AI-enhanced education existed before 2022 (e.g., learning analytics, adaptive tutoring), we treat late 2022 as a pragmatic boundary in this study because the public release and rapid uptake of GenAI tools introduced a step-change in the ease of generating assessable outputs, intensifying concerns around authorship, integrity, and what constitutes ‘authentic’ learning.

In this review, authenticity is conceptualised as a multi-dimensional pedagogical construct rather than as a simple real-world simulation. It encompasses (a) authentic interaction, characterised by unscripted, face-to-face social engagement; (b) authentic learning contexts, shaped by embodied, material, and social constraints; and (c) authentic assessment signals, where evidence of learning is embedded in process, participation, and decision-making. These dimensions are particularly salient in the context of GenAI, as they foreground aspects of learning that are difficult to externalise or automate. This conceptualisation also aligns with established perspectives on authentic learning and assessment, which emphasise meaningful social interaction, situated activity, and process-oriented evidence of learning rather than decontextualised task completion (Herrington et al., 2010; Ashford-Rowe et al., 2014).

This development has triggered intense debate about how to sustain authentic learning, critical thinking, and social interaction in an era of automated content creation. In response, educators have begun to re-examine tactile and collaborative pedagogies that emphasise human judgement and creativity, qualities not easily simulated by algorithms (Knox, 2023). Analogue games, by virtue of their embodied, dialogic, and context-dependent nature, may represent a form of pedagogical counterbalance to GenAI-mediated instruction. They situate learning within physical, interpersonal spaces where meaning emerges through conversation and negotiation rather than machine-generated output.

For this reason, 2022 serves as a meaningful analytical boundary in this review. The year marks a pedagogical inflection point: pre-2022 studies reflect a digital-expansion era, while post-2022 research occurs in a context of GenAI disruption and reflexive re-evaluation. Categorising studies as pre-GenAI (2010–2022) or post-GenAI (2023–2025) allows exploration of whether the emergence of generative technologies has coincided with renewed scholarly attention to analogue forms of active learning.

### 1.4. Rationale for a Scoping Review

A scoping review methodology is particularly appropriate for this topic, given the breadth and heterogeneity of the literature on analogue games in higher education. Scoping reviews enable researchers to map existing evidence, clarify conceptual boundaries, and identify knowledge gaps without imposing the restrictive quality criteria of a systematic review (Arksey & O’Malley, 2005; Munn et al., 2018). This approach is well suited to emerging pedagogical fields where empirical and descriptive studies coexist across multiple disciplines and publication types.

No previous review, to our knowledge, has focused exclusively on non-digital games within higher education, nor has any examined how their pedagogical role may be evolving in the context of GenAI. Existing meta-analyses of game-based learning often aggregate digital and analogue interventions, obscuring the unique social and tactile dimensions of physical play. This scoping review therefore contributes an original synthesis of the ways in which traditional games are being used as active learning strategies in contemporary tertiary education.

### 1.5. Aims and Research Questions

The aim of this review is to map and synthesise published evidence on the use of traditional, non-digital games, specifically board and card formats, as active learning strategies in higher education. The review addresses the following research questions:

1. In what disciplinary contexts are non-digital games being used as active learning tools in tertiary education?
2. What learning outcomes, cognitive, affective, behavioural, or social, are reported?
3. What types of analogue games and pedagogical designs are most commonly employed?
4. Has the emergence of GenAI since 2022 acted as a driver or brake on these analogue pedagogies?

By addressing these questions, the review seeks to illuminate how analogue game-based learning contributes to active, human-centred education at a time when automation and artificial intelligence are reshaping the boundaries of teaching and learning.

### 1.6. Structure of the Paper

The remainder of this paper presents the methods, results, and discussion of the review. Section 2 outlines the search strategy, inclusion criteria, and data-charting procedures. Section 3 reports descriptive findings on disciplinary distribution, game types, learning outcomes, and temporal trends. Section 4 discusses the implications of these findings for pedagogy in the GenAI era, highlighting how analogue games may support authenticity, creativity, and collaboration in higher education.

## 2. Methodology

### 2.1. Review Design

This study employed a scoping review approach following the methodological framework of Arksey and O’Malley (2005), refined by Levac et al. (2010), and reported in accordance with the Preferred Reporting Items for Systematic Reviews and Meta-Analyses extension for Scoping Reviews (PRISMA-ScR) (Tricco et al., 2018). Scoping reviews are appropriate for mapping heterogeneous bodies of literature and identifying conceptual boundaries within an emerging field (Munn et al., 2018).

The review aimed to systematically identify and analyse peer-reviewed studies that examine the use of traditional, non-digital games, such as board and card games, as active learning strategies in tertiary or higher-education settings. A subsidiary objective was to explore whether the rise in GenAI since late 2022 has acted as a driver or brake on the uptake of such analogue pedagogies.

### 2.2. Research Questions

The review was guided by the overarching question:

- How are traditional, non-digital games used as active learning strategies in tertiary or higher-education contexts, what learning outcomes are reported, across which disciplines, and in what ways, if any, is GenAI influencing these pedagogical practices?

The sub-questions were:

- In what disciplinary domains are non-digital games applied?
- What learning outcomes (knowledge, skills, attitudes, engagement) are reported?
- What types of games and active-learning designs are used?
- Has the emergence of GenAI (2022–2025) been identified as a contextual driver or barrier?

### 2.3. Search Strategy

A comprehensive search was conducted in October 2025 using the Scopus database, selected for its broad coverage of educational, behavioural, and applied sciences literature. The search syntax was designed to capture studies referring to analogue, non-digital games used for active learning in higher education:

(TITLE-ABS-KEY(“board game*” OR “card game*” OR “tabletop game*” OR “analog game*”))

AND (TITLE-ABS-KEY(“active learning” OR “experiential learning” OR “gamification”))

AND (TITLE-ABS-KEY(“higher education” OR universit* OR college OR “tertiary education”))

Although the intent of the study was to focus only on card and board games, the search terms “tabletop game*” and “analog game*” were initially adopted to be inclusive, but any documents which did not specifically include card and/or board games were later excluded.

The search filters applied were: Date range: 2010–2025; Language: English; Document type: peer-reviewed articles, conference papers, and book chapters.

The database choice was Scopus, due to its broad multidisciplinary and international coverage.

### 2.4. Eligibility Criteria

Studies were eligible if they met all of the following criteria:

- Described the design, implementation, or evaluation of a non-digital game (e.g., board, card) used for educational purposes;
- Were conducted within a tertiary or higher-education context (undergraduate, postgraduate, or faculty development);
- Explicitly situated the activity within an active learning, experiential, or gamified pedagogy;
- Were published in English between 2010 and 2025;
- Provided sufficient empirical or descriptive detail to extract pedagogical outcomes.

Exclusion criteria were:

- Digital, computer, or video-game interventions;
- Non-higher education, community, or informal education settings;
- Papers lacking educational or pedagogical analysis;
- Non-English texts;
- Records where full-text access was unavailable.

### 2.5. Screening and Selection Process

The initial search returned 2480 records. All results were exported from Scopus in CSV format and imported into Microsoft Excel for organisation and cleaning. To support the initial screening process, automated keyword filtering was employed as a screening aid rather than as a mechanism for final inclusion or exclusion decisions. Keywords commonly associated with digital or technology-mediated learning environments (e.g., “online,” “virtual,” “app,” “computer-based,” “video game,” “mobile,” “VR”) were used to flag records that were likely to fall outside the scope of analogue, non-digital game-based learning. Records containing such terms were not automatically excluded but were instead identified for closer manual review.

All records flagged through automated filtering were subsequently reviewed manually against the inclusion and exclusion criteria to ensure that potentially relevant studies were not inadvertently removed. Automated columns flagged the presence of terms such as “digital,” “higher education,” “active learning,” “board,” and “card”. This enabled the rapid removal of clearly irrelevant or digital-only studies. Manual screening of titles and abstracts then confirmed relevance to the inclusion criteria.

Where eligibility could not be clearly determined based on title and abstract screening, records were retained for full-text review. This iterative approach was used to minimise the risk of excluding studies that may have addressed analogue game-based learning despite ambiguous terminology or overlapping references to digital contexts.

**Figure 1:**
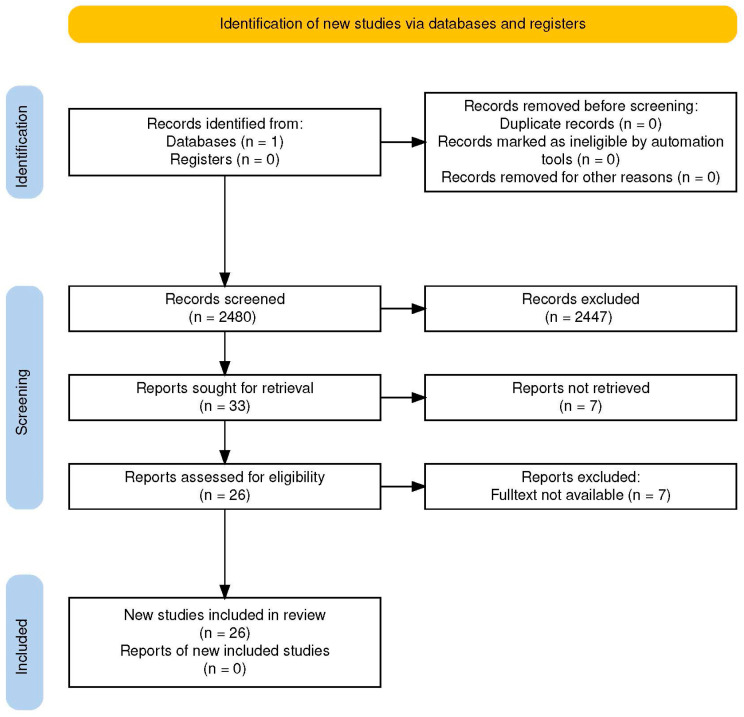
PRISMA-ScR Flow Diagram. PRISMA-ScR flow diagram illustrating the identification, screening, and inclusion of studies. From an initial 2480 records retrieved through Scopus, 33 full-text papers were assessed for eligibility, and 26 studies met all inclusion criteria. Adapted from Tricco et al. (2018).

Full-text screening verified accessibility and educational focus. Reasons for exclusion were recorded (e.g., “digital-only,” “school-level,” “no active-learning component”). A total of 33 papers proceeded to full-text review; after deduplication and relevance checks, 26 unique studies met all inclusion criteria. These were retained for data charting and synthesis (Figure 1).

### 2.6. Data Charting and Extraction

Consistent with established scoping review methodology, no formal methodological quality appraisal of included studies was conducted. Rather than evaluating studies for comparative effectiveness or risk of bias, the purpose of this review was to map the characteristics, contexts, and reported outcomes of analogue game-based learning in higher education. For scoping reviews, the primary aim is to systematically map the breadth, range, and nature of evidence on a topic, rather than to critically appraise individual studies or synthesise effect sizes as in systematic reviews. Scoping review frameworks, including the foundational work by Arksey and O’Malley (2005) and subsequent reporting guidance such as PRISMA-ScR (Tricco et al., 2018), emphasise this mapping and descriptive focus rather than formal quality assessment.

A structured data-charting framework was used to guide data extraction and synthesis. This framework defined the analytic categories used to systematically capture information from each included study, supporting descriptive analysis and thematic mapping rather than outcome comparison. These predefined categories functioned as an analytic framework for organising and synthesising the literature, allowing patterns and gaps in the use of analogue game-based learning to be identified across diverse educational contexts.

Data from the 26 included studies (see Appendix A) were extracted using a standardised Excel charting template. Each entry captured the following fields:

- Author(s), year, and DOI
- Country and disciplinary context
- Game type (board, card, hybrid)
- Educational level (undergraduate, postgraduate, faculty)
- Study design or evaluation method
- Reported learning outcomes (knowledge, skills, engagement, attitudes)
- Pedagogical framing (active learning, experiential, problem-based, gamified)
- Reference to GenAI or post-2022 contextual discussion

Coding was iterative. Descriptive frequency analysis was used to summarise disciplines, game modalities, and outcome categories. Temporal grouping distinguished pre-GenAI (2010–2022) and post-GenAI (2023–2025) studies to identify potential shifts in analogue pedagogy emphasis.

### 2.7. Data Analysis and Synthesis

The analysis was descriptive and thematic, consistent with scoping-review methodology. Quantitative descriptors (discipline, year, game type, outcome direction) were tabulated. Qualitative content analysis of author discussions and conclusions identified recurring themes such as engagement, collaboration, conceptual understanding, and authenticity. Studies were compared by discipline to map where analogue game-based learning is most prevalent and by publication year to assess any temporal clustering around the GenAI era. Although no studies directly examined GenAI, post-2023 publications often referenced AI as a contextual driver of renewed interest in embodied and social learning, positioning non-digital games as counterbalancing tools within increasingly digitised learning environments.

### 2.8. Limitations

While the scoping review corpus was derived from a predefined Scopus search strategy (selected due to its multidisciplinary coverage), additional literature indexed in ERIC and Web of Science was consulted to strengthen the conceptual framing and discussion, and to reduce some potential bias. The focus on English-language publications may underrepresent work in non-English contexts. As a scoping rather than a systematic review, no formal quality appraisal was performed. Nevertheless, the transparent search strategy, explicit inclusion criteria, and structured data-charting process provide a robust mapping of current scholarship on non-digital game-based learning in higher education.

### 2.9. Ethical Considerations

All data were derived from published sources and publicly accessible materials.

No ethical approval was required.

## 3. Results

### 3.1. Overview of Included Studies

The final dataset comprised 26 unique studies published between 2012 and 2025, representing a steady but uneven distribution of research into analogue game-based learning in higher education. Publication frequency increased notably after 2020, with a modest surge observed in 2023–2025, coinciding with the public introduction of GenAI.

Of the 26 studies, 12 were published before 2023 (pre-GenAI) and 12 from 2023–2025 (post-GenAI). Most were journal articles (n = 19), supplemented by conference papers (n = 5) and book chapters (n = 2). Geographically, the research was highly international, spanning Europe, North America, Asia, and South/Latin America.

Study designs were predominantly case studies or classroom evaluations (n = 17), followed by design-based research (n = 5), quasi-experimental designs (n = 3), and conceptual or framework papers (n = 1). Sample sizes ranged from small cohorts (10–30 students) to large classes exceeding 200 participants. Despite methodological diversity, nearly all studies reported positive or partially positive outcomes for learning, engagement, or motivation.

### 3.2. Disciplinary Distribution

Analogue game-based learning has been implemented across a remarkably wide range of disciplines (Table 1). The most frequent domains were STEM subjects (n = 11), particularly chemistry, engineering, and environmental science, followed by business and management (n = 5), health and medical education (n = 4), social sciences and education (n = 4), and arts or civic learning (n = 2).

Across disciplines, the common rationale for adopting analogue games was to increase engagement, foster collaboration, and bridge theory–practice divides. In STEM and business contexts, games were often used to simulate systems (e.g., project management, resource allocation, molecular interactions), while in health and social science courses, they were used to encourage ethical reasoning, empathy, or reflective practice.

A smaller subset of studies, particularly post-2023, explicitly linked analogue gameplay to authentic learning or academic integrity concerns arising in AI-mediated education (e.g., Conway & Smith, 2026; Thammaboosadee, 2025).

### 3.3. Game Types and Pedagogical Designs

The most common game modalities were board games (n = 15), followed by card games (n = 6), hybrid analogue–digital formats (n = 3), and mixed format where cards/boards were part of the games (n = 2).

Board games predominated in engineering, sustainability, and management education, where learners assumed collaborative roles to solve contextualised problems (e.g., DOMEGO; Taillandier et al., 2021). Card games were more common in biochemistry, health, and business ethics, often used for concept reinforcement or scenario discussion (e.g., Amino-Structure, Gómez-Buitrago et al., 2024; Clinical Coaching Cards, Watsjold & Zhong, 2020).

Hybrid games integrating limited digital components (e.g., Yeo, 2021) blurred the analogue–digital boundary, but retained the tactile, group-based structure that distinguishes non-digital play. Across designs, the learning mechanics most frequently identified were role assumption, problem-solving, and peer discussion, hallmarks of active and experiential pedagogy.

### 3.4. Reported Learning Outcomes

All included studies reported at least one positive learning outcome, though emphasis varied by discipline. Common categories are summarised in Table 2.

Although measurement varied, most studies used self-report surveys or qualitative feedback. Fewer employed formal statistical analysis, though several noted significant pre-post gains (e.g., Rafiq et al., 2025, *p* < 0.001). A recurring theme across disciplines was that analogue games provide ‘safe spaces’ for experimentation, allowing learners to make and correct mistakes through play.

### 3.5. Temporal Trends: Pre- and Post-GenAI

When viewed chronologically, the dataset reveals subtle but meaningful shifts in pedagogical framing. Between 2010 and 2022, most studies positioned analogue games as alternative engagement tools within broader digital or blended learning movements. Their focus was primarily motivational, addressing student disengagement, conceptual difficulty, or low participation in lecture-based courses.

By contrast, in the post-GenAI period (2023–2025), several studies began to frame non-digital play as a pedagogical counterbalance to automation and AI-mediated learning. For instance, Conway and Smith (2026) described card-based business simulations as “re-humanising” teamwork and ethical reflection in a data-driven era, while Thammaboosadee (2025) used a drama game to cultivate empathy and civic agency. These newer studies often invoked the language of authenticity, social presence, and embodied cognition, suggesting a subtle conceptual reorientation from novelty to necessity in maintaining human interaction within AI-rich environments.

### 3.6. Synthesis of Emerging Themes

Across the literature sample, three integrative themes emerge:

1. Embodied Engagement: Analogue games foster physical and social participation, enabling learners to externalise reasoning and negotiate meaning collaboratively. This embodiment contrasts with the increasingly disembodied modes of digital and AI-mediated learning.
2. Authenticity and Agency: Post-2023 studies emphasise authenticity, trust, and ethical decision-making, framing analogue games as contexts where human judgement and unpredictability are valued.
3. Pedagogical Resilience in the GenAI Era: Rather than being supplanted by technology, analogue game-based learning appears to have gained renewed pedagogical legitimacy as educators seek resilient, human-centred methods that complement AI tools without replicating them.

In summary, analogue games remain a vibrant, adaptable component of active learning in higher education. The evidence base, while modest, spans diverse disciplines and consistently highlights improvements in engagement, collaboration, and understanding. Although few studies address GenAI directly, the timing and discourse of recent publications imply a pedagogical recalibration: as generative technologies permeate the learning environment, analogue games are being re-valued for their capacity to sustain human creativity, dialogue, and ethical reasoning within a rapidly digitalising academy.

## 4. Discussion

### 4.1. Overview

This scoping review sought to map how non-digital or analogue games, such as board and card formats, are used as active learning strategies in tertiary education, what outcomes are reported, across which disciplines, and whether GenAI acts as a driver or brake on these pedagogies.

The synthesis of 26 studies reveals that analogue game-based learning (GBL) continues to occupy a distinctive and valuable niche within higher education.

While digital tools dominate contemporary discourse, the persistence and recent resurgence of non-digital games illustrate a continuing demand for embodied, social, and authentic forms of learning that resist automation and foster meaningful engagement.

### 4.2. Analogue Games as Embodied and Social Pedagogies

Across disciplines, analogue GBL demonstrates a shared commitment to embodied learning, where knowledge is constructed through physical interaction, dialogue, and reflection. The tactile nature of cards, boards, and tokens transforms learning from a cognitive process into a social event, facilitating participation and inclusivity (Gee, 2008; Nicholson, 2015). The emphasis on embodied and socially situated activity in analogue game-based learning reflects broader educational theories that position learning as experiential and participation-based rather than solely cognitive or representational (Kolb, 2015; Lave & Wenger, 1991).

Studies in engineering and business education (Taillandier et al., 2021; Conway & Smith, 2026) repeatedly emphasised that the material and collaborative aspects of gameplay encouraged teamwork and collective reasoning in ways that digital simulations did not. Similarly, in medical and health contexts (Watsjold & Zhong, 2020; Rafiq et al., 2025), card-based learning fostered reflection and interpersonal communication, skills increasingly recognised as essential in professional practice.

The concept of embodiment aligns with constructivist and socio-cultural theories of learning (Vygotsky, 1978), underscoring that knowledge emerges through participation and negotiation. Analogue games therefore contribute to a community of practice (Lave & Wenger, 1991) that integrates knowledge, skill, and social presence, qualities that risk erosion in purely digital or AI-mediated settings.

### 4.3. Learning Outcomes and Pedagogical Value

The reviewed literature consistently reports high levels of engagement, motivation, and conceptual understanding, confirming the pedagogical robustness of analogue play. Quantitative and qualitative data alike reveal that students find non-digital games enjoyable, relevant, and memorable. In many cases, they function as boundary objects, linking abstract theoretical content to real-world contexts (Star & Griesemer, 1989). For example, biochemistry-based card games (Gómez-Buitrago et al., 2024) helped visualise molecular structures, while environmental management games (Kurisu et al., 2021) translated complex sustainability concepts into tangible decision-making scenarios. In professional education, games often supported reflective practice and ethical reasoning (Babl et al., 2025), echoing calls for whole-person learning approaches (Kolb, 2015).

While most studies used self-report data (post-session perception surveys or reflective feedback) rather than controlled experiments (for example, Huang & Levinson, 2012, Conway & Smith, 2026), the consistency of positive outcomes across contexts provides a compelling body of evidence for analogue games as legitimate active learning tools, not mere novelties. However, whilst these approaches support conclusions about engagement, they may limit causal claims about long-term knowledge gains.

### 4.4. The Influence of GenAI: Catalyst, Not Competitor

Although none of the studies directly evaluated GenAI applications, temporal and discursive patterns suggest that its emergence has indirectly revitalised interest in non-digital, human-centred pedagogies. From 2023 onwards, several studies explicitly referenced the need for authentic, interactive, and socially grounded learning environments in the face of AI’s growing capacity to automate knowledge reproduction (Conway & Smith, 2026; Thammaboosadee, 2025).

This reflects a broader pedagogical anxiety about the potential erosion of originality, dialogue, and critical thinking in AI-mediated learning spaces (Kasneci et al., 2023; Knox, 2023). In this sense, GenAI can be viewed not as a competitor to analogue pedagogy, but as a catalyst for rebalancing. Educators are reasserting the value of learning experiences that are totally human: those involving physical presence, moral reasoning, humour, and serendipity. Analogue games provide precisely this: they make thinking visible, invite disagreement, and depend on mutual accountability. In doing so, they help preserve the social contract of education, positioning students as co-constructors rather than consumers of knowledge.

This finding resonates with theoretical perspectives on post-digital education (Jandrić et al., 2018), which propose that effective pedagogy now involves navigating both digital and analogue affordances rather than privileging one over the other.

The re-emergence of board and card games may therefore represent an act of pedagogical resilience: an adaptive strategy that maintains engagement and authenticity amidst rapid technological change.

### 4.5. Disciplinary Breadth and Transferability

One striking outcome of this review is the disciplinary diversity of analogue GBL.

While STEM subjects dominate, applications in business, health, and the arts reveal its flexibility and cross-curricular potential. In each context, the analogue medium was valued for different reasons:

- In STEM, to model systems and encourage experimentation;
- In business and economics, to develop strategic thinking and risk management;
- In health, to facilitate communication and empathy;
- In arts and civics, to provoke dialogue and ethical reflection.

This breadth suggests that analogue GBL functions less as a discipline-specific method and more as a pedagogical stance: one that prioritises interaction, creativity, and reflection regardless of subject matter. Such versatility reinforces its relevance within multidisciplinary curricula and graduate employability frameworks, emphasising collaboration, adaptability, and problem-solving.

The findings of this review can also be interpreted in the wider context of higher education’s response to the emergence of GenAI. As GenAI tools increasingly automate information retrieval and textual production, they challenge traditional conceptions of learning and assessment that prioritise knowledge recall and written output (Kasneci et al., 2023; Topali et al., 2025). Within this shifting landscape, active learning has acquired renewed significance as a means of safeguarding human-centred dimensions of education, including collaboration, creativity, and ethical reasoning (Knox, 2023; Luckin et al., 2016). The sustained use of non-digital games across disciplines suggests that educators are seeking pedagogical approaches which reintroduce uncertainty, dialogue, and shared agency into learning processes that might otherwise become technologically mediated. Viewed in this way, analogue play does not simply persist in spite of AI innovation but evolves as a complementary practice, one that reinforces the importance of embodied, social, and reflective engagement in cultivating the higher-order capacities that define meaningful learning in the post digital era.

### 4.6. Gaps and Opportunities

Despite promising results, the evidence base remains fragmented and uneven. Few studies employed rigorous experimental designs, longitudinal follow-up, or comparative analysis against digital or lecture-based instruction. Moreover, the GenAI context remains underexplored: while recent papers acknowledge its pedagogical implications, none have empirically examined how analogue and AI-enhanced approaches might coexist within blended curricula.

There is a clear need for future research to investigate how analogue games might complement AI tools, for instance, using GenAI to design or debrief physical games, or to support reflection after in-person play. Equally, few studies addressed inclusion or accessibility, despite analogue games’ potential to reduce technological barriers. Further work could examine how these methods engage diverse learners, support neurodiverse students, or foster belonging in hybrid and remote contexts.

### 4.7. Reframing Active Learning in the GenAI Era

Taken together, the findings suggest that analogue games are part of a broader movement towards re-humanising education in the GenAI era. As automation accelerates, active learning may increasingly depend on cultivating embodied presence, creativity, and social reasoning, competencies least amenable to machine replication. Analogous to earlier pedagogical responses to industrial or digital revolutions, non-digital play functions as both resistance and renewal: resisting the over-instrumentalization of learning, while renewing its affective and communal dimensions.

This shift reflects a reframing of “active learning” itself, from a focus on activity and engagement to one of authentic human participation in meaning-making. As recent scholarship on academic integrity and assessment in the context of GenAI similarly highlights, there is a need for assessment approaches that foreground process, participation, and situated performance rather than easily externalised outputs (Boud & Falchikov, 2006; Eaton, 2023).

In this reframed paradigm, analogue games are not nostalgic artefacts but timely instruments for cultivating the cognitive, emotional, and ethical capacities needed in an AI-saturated world.

## 5. Conclusions

This scoping review set out to explore how traditional, non-digital games, specifically board and card formats, are employed as active learning strategies in higher education, what outcomes they produce, and whether the emergence of GenAI has influenced their pedagogical role.

Across 26 studies published between 2010 and 2025, the evidence demonstrates that analogue game-based learning (GBL) remains a vital, flexible, and enduring approach to fostering engagement, understanding, authenticity, and collaboration in tertiary education. Rather than displacing such approaches, AI appears to have prompted educators to revalue the irreplaceably human aspects of play, dialogue, and creative problem-solving.

Analogue games were found across a diverse range of disciplines, from chemistry and engineering to business, health, and the arts. Despite their variety, these interventions share common pedagogical strengths: they create embodied, social spaces where learners co-construct knowledge, practise decision-making, and engage authentically with complex ideas. Such qualities have become especially valued in a post-2022 educational landscape increasingly mediated by AI technologies. Hence, analogue games represent not an anachronism, but a living, adaptive pedagogy, anchoring learning in human interaction in this era where the boundaries between human and machine intelligence are being redrawn.

While no included study directly assessed the use of GenAI, post-2023 publications reveal a shift in discourse, framing analogue play as a means of restoring human interaction, authenticity, and ethical reasoning within technology-rich learning environments. In this sense, GenAI appears less as a competitor than as a contextual catalyst, renewing attention to pedagogical designs that foreground creativity, empathy, and dialogue.

From a practical standpoint, this review affirms the relevance of non-digital games for educators seeking active, low-cost, and inclusive methods adaptable across disciplines. Their tactile and interpersonal nature aligns with constructivist principles and can complement, rather than replace, digital or AI-enhanced learning.

Institutions exploring AI integration might therefore view analogue play not as an alternative but as a counterbalancing partner that sustains learner agency, social connection, and critical engagement.

Future research should build on these insights through comparative and longitudinal designs that examine learning gains across modalities and time.

In particular, studies could explore hybrid approaches that combine analogue play with GenAI-supported reflection, scenario generation, or adaptive feedback.

Such work would contribute to a more holistic understanding of how analogue and AI-mediated pedagogies might coexist within post-digital education.

In conclusion, analogue games remain pedagogically resilient in the age of artificial intelligence. They remind educators that while technologies evolve, the heart of learning, curiosity, collaboration, and creativity, remains distinctly human.

## Figures and Tables

**Table 1:** Studies by discipline and game type. Characteristics of included studies (n = 26) examining non-digital games as active learning strategies in higher-education settings, grouped by discipline and game type.

Discipline	Number of Studies	Typical Game Type	Example Studies
STEM (chemistry, engineering, environmental science)	11	Board/card	(Babl et al., 2025; Taillandier et al., 2021; Kurisu et al., 2021)
Business/Economics	5	Card/strategic board	(Conway & Smith, 2026; Marcos & López-García, 2024)
Health/Medical	4	Card/board	(Watsjold & Zhong, 2020; Rafiq et al., 2025)
Education/Social Sciences	4	Board/mixed	(Ferreira Dias et al., 2024; Sierra & Suárez-Collado, 2021)
Arts/Humanities/Civic	2	Mixed	(Thammaboosadee, 2025)

**Table 2:** Outcomes by learning category. Summary of learning outcomes and thematic patterns from the included studies (n = 26).

Learning Outcome Category	Frequency	Illustrative Examples
Engagement and motivation	22	“Students expressed higher enthusiasm and persistence” (Ferreira Dias et al., 2024)
Conceptual or procedural understanding	17	Very effective as an introduction to taphomony” (Martindale & Weiss, 2020)
Collaboration and communication	15	“Enhanced peer-to-peer explanation and teamwork” (Rafiq et al., 2025)
Critical thinking/ethical reasoning	8	“Prompted discussion of professional dilemmas” (Babl et al., 2025)
Reflection and metacognition	6	“Facilitated self-assessment and confidence” (Watsjold & Zhong, 2020)
Creativity/problem-solving	5	“Encouraged innovative solutions under constraint” (Thammaboosadee, 2025)

## Data Availability

Data are contained within the article.

## Abbreviations

The following abbreviations are used in this manuscript:

AI	Artificial Intelligence
GBL	Game-based Learning
Gen-AI	Generative Artificial Intelligence
PRISMA-ScR	Preferred Reporting Items for Systematic Reviews and Meta-Analyses extension for Scoping Reviews

## Appendix A. Included Studies (n = 26)

Babl, R.M., Cole, K.D., Parish, R.L., Wartman, C., Howington, D., MacSorley, R., Adcock, K.G. (2025). Promoting values and ethics in collaborative health-care training. *Journal of the American College of Clinical Pharmacy*, *8*, 732–740. https://doi.org/10.1002/jac5.70072.
Conway, E., & Smith, R. (2026). Playing with purpose: A game-based approach to teaching business metrics. *International Journal of Management Education*, *24*(1), 101310. https://doi.org/10.1016/j.ijme.2025.101310.
Cruza, I. A., (2025). Educational Impact of Infinity Masters: A Digital Board Game on Arithmetic, Strategy, and Interactive Learning. *International Journal of Computer Games Technology*, (1), 2470446. https://doi.org/10.1155/ijcg/2470446.
Ferreira Dias, M., Alves, C., Amorim, M. (2024). Board games and entrepreneurial skills acquisition. In T. Connolly (Ed.), *Proceedings of the European conference on game-based learning (ECGBL)* (pp. 135–144). https://doi.org/10.34190/ecgbl.18.1.2666.
Gómez Buitrago, P. Tobar-Muñoz, H., Arteaga, D. (2024). Amino-Structure: A card game for amino-acid learning in biochemistry classes. *Journal of Chemical Education*, *101*(5), 2141–2148. https://doi.org/10.1021/acs.jchemed.3c01209.
Huang, A., & Levinson, D. (2012). To game or not to game: Teaching transportation planning with board games. *Transportation Research Board*, *2307*(1), 141–149. https://doi.org/10.3141/2307-15.
Jaroenkhasemmeesuk, C. Lima, R. M., Horgan, K. Mesquita, D. Supeekit, T. (2023). Active learning in engineering education: Case study in mechanics. In Pisut Koomsap, Adam Cooper, Josip Stjepandić (Eds.), *Leveraging Transdisciplinary Engineering in a Changing and Connected World*, *41*, 633–642. https://doi.org/10.3233/ATDE230659.
Kurisu, K., Okabe, H., Nakatani, J., Moriguchi, Y. (2021). Development of board game to encourage life-cycle thinking. *Cleaner and Responsible Consumption*, *3*, 100033. https://doi.org/10.1016/j.clrc.2021.100033.
Makri, E. G. (2022). Does the cards against calamity learning game facilitate attitudes toward negotiation, civics, and sustainability? empirical findings from greek graduates. *Education Sciences*, *12*(11), 738. https://doi.org/10.3390/educsci12110738.
Marcos, S., & López-García, B. (2024). Boardcraft: Learning the art of strategic decision-making card by card. *Proceedings of the European conference on game-based learning (ECGBL)*, *18*, *1* 1047–1051. https://doi.org/10.34190/ecgbl.18.1.2646.
Martindale, R. C., & Weiss, A. M. (2020). ”Taphonomy: Dead and fossilized”: A new board game designed to teach college undergraduate students about the process of fossilization. *Journal of Geoscience Education*, *68*(3), 265–285. https://doi.org/10.1080/10899995.2019.1693217.
Rafiq, R., Matthews, H., Abedalreza, D., Yahya, F., Jones, M.A. (2025). Card games as effective tools to enhance foundation-year health and safety inductions. *FEBS Open Bio*, *15*(12), 2080–2095. https://doi.org/10.1002/2211-5463.70140.
Ruiz-Arroyo, M., Castillo, A., & Gutierrez, L. (2025). Active Learning and Authentic Assessment Through Gamification: A Board Game Experience in Eliseo Vilalta-Perdomo, Alessandra Scroccaro, David Ernesto Salinas-Navarro, Rosario Michel-Villarreal (Eds.) *The Emerald Handbook of Active Learning For Authentic Assessment*, 125–146. https://doi.org/10.1108/978-1-83797-857-120251007.
Sarinho, V.T. (2019). *Masters of the Process: A Board Game Proposal for Teaching Software Management and Software Development Process*. Proceedings of the XXXIII Brazilian Symposium on Software Engineering, 532–536. https://doi.org/10.1145/3350768.3352459.
Selamat, A.I., & Ngalim, S.M. (2022). Putra Salamanis board game: the game of bookkeeping for fundamental financial accounting learning. *Accounting Education*, *31*(5), 596–614. https://doi.org/10.1080/09639284.2021.2015408.
Shemran, R.P., Clark, R.M., Bilec, M.M., Landis, A.E., Parrish, K. (2017). *Developing a framework to better engage students in STEM via game design: Findings from year 1*. American Society for Engineering Education Annual Conference and Exposition, Conference Proceedings. Jun 24 2017, 20435. https://peer.asee.org/27702.
Sierra, J., & Suárez-Collado, A. (2021). The transforming generation: Economic decisions and sustainability. *International Journal of Sustainability in Higher Education*, *22*(5), 1087–1107. https://doi.org/10.1108/IJSHE-06-2020-0221.
Sotoca-Orgaz, P., Arevalo-Baeza, M., Perez-Lopez, I.J. (2025). Impact of a serious board game on future physical education teachers. *International Journal of Serious Games*, *12*(3), 149–169. https://doi.org/10.17083/mt8e7w68.
Szilagyi, S., Korei, A., Vaiciulyte, I. (2025). The Role of Non-Digital and Digital UNO-Type Card Games as Learning Media in Different Levels of Mathematics Education: A Systematic Review. *Education Sciences*, *15*(8), 1030. https://doi.org/10.3390/educsci15081030.
Taillandier, F., Micolier, A., Sauce, G., Chaplain, M. (2021). DOMEGO: A board game for learning how to manage a construction project. *International Journal of Game-Based Learning*, *11*(2), 20–37. https://doi.org/10.4018/IJGBL.2021040102.
Thammaboosadee, R. (2025). Stardust Odyssey: City’s Last Stand—Utilising Process Drama and Design-Based Research in Tabletop Game Workshops to Reimagine Active Citizenship in Thailand’s Neoliberal Education Context. *Designs for Learning*, *16*(1), 22–35. https://doi.org/10.16993/dfl.235.
Thammavongsy, Z., Morris, M.A., & Link, R.D. (2020). 1H NMR Spectrum: A Team-Based Tabletop Game for Molecular Structure Elucidation. *Journal of Chemical Education*, *97*(12), 4385–4390. https://doi.org/10.1021/acs.jchemed.0c01267.
Tzioutzios, D., Song, S., Bean, H., Chabay, I., Cruz, A.M. (2024). Serious gaming for Natech risk awareness: Introducing EGNARIA. *International Journal of Disaster Risk Reduction*, *115*, 105080. https://doi.org/10.1016/j.ijdrr.2024.105080.
Watsjold, B., & Zhong, D. (2020). Clinical coaching cards: A game of active learning theory and teaching techniques. *MedEdPORTAL*, *16*(1), 11042. https://doi.org/10.15766/mep_2374-8265.11042.
Wu, J. H., Su, P. H., Wu, H. Y., Hsin, Y. M., Lin, C. H., & Lee, C. Y. (2025). Educational board game for training dental and dental hygiene students in patient safety issues. *BMC Medical Education*, *25*, 518. https://doi.org/10.1186/s12909-025-07284-7.
Yeo, D. (2021). nCoV-BusterBot: Mission Simulation Lab Modules for Supporting a Lab-based Autonomous Systems Class in a Remote Learning Environment. *AIAA SciTech Forum*, *January 2021*. https://doi.org/10.2514/6.2021-0040.
